# Exploring SARS-CoV-2 impact on blood-brain barrier and its composition: A review

**DOI:** 10.1097/MD.0000000000046093

**Published:** 2025-11-21

**Authors:** Jin Li, Shangfu Xu, Sixu Guo

**Affiliations:** a Provincial Department of Education, Key Laboratory of Infectious Disease & Biosafety, Zunyi Medical University, Zunyi, Guizhou, China; b Key Laboratory of Cell Engineering of Guizhou Province, Affiliated Hospital of Zunyi Medical University, Zunyi, Guizhou, China.

**Keywords:** astrocytes, BBB, brain microvascular endothelial cells, COVID-19, microglia, SARS-CoV-2

## Abstract

Inflammatory responses including glial activation, and upregulated inflammatory factors occurred after severe acute respiratory syndrome coronavirus 2 (SARS-CoV-2) infected central nervous system. Blood-brain barrier (BBB) disruption has been implicated in coronavirus disease 2019 (COVID-19) pathogenesis and may predispose to the long-lasting neurological damage even after the epidemic ends. The BBB is a highly selective dynamic interface to protects the brain from neurotoxins and the elimination of byproducts of brain metabolism via efflux transporters. The COVID-19 pandemic has introduced new challenges in managing neurological conditions, and understanding SARS-CoV-2 journey through BBB and the interconnections between the members of BBB is crucial. This review aims to summarize and elucidate the damage to the main constituent cells of BBB, including brain microvascular endothelial cells, astrocytes, and microglia and its contribution to COVID-19. Further understanding of these interactions may facilitate the development of improved treatment options and preventative measures of central nervous system injury due to COVID-19.

## 1. Introduction

The global pandemic of coronavirus disease 2019 (COVID-19), caused by severe acute respiratory syndrome coronavirus 2 (SARS-CoV-2), which belong to the third member of the coronavirus family, due to its high morbidity and mortality rate, has infected over 619 million patients and resulted in 6.55 million deaths worldwide until August 2022.^[1]^ As COVID-19 is primarily a respiratory disease, the majority of COVID-19 patients display respiratory symptoms include sore throat, cough, fever or chills, shortness of breath and so on, which manifest can range from mild to life-threatening.^[2]^ Although the main clinical manifestations of COVID-19 are associated with respiratory symptoms, reports of neurological signs and symptoms are increasing.^[3]^ There are early warnings that the function of the nervous system, especially the central nervous system, will be significantly impaired when infected by the virus and a wide range of neurological manifestations is reported in association with COVID-19, including cerebral metabolic dysfunction, headache, neurodegeneration, cognitive deficits and even brain stem encephalitis, which called Long COVID.^[4,5]^ Understanding the basis of such consequences, it is helpful to examine the interrelationship between “COVID-19 effects” and neurological disorders, as well as lead to new therapeutic strategies for evolving the impact of the virus on the central nervous system. Here a comprehensive literature search was performed in PubMed using different combinations of keywords such as “COVID-19,” “SARS-CoV-2,” “the central nervous system,” “blood-brain barrier,” “brain microvascular endothelial cells (BMECs),” “astrocytes,” “microglia,” and “exosomes.” Articles published between 2020 and 2025 were included. Additional studies were identified by manually screening reference lists of relevant reviews. Studies were included if they focused on clinical outcomes of COVID-19, and were peer-reviewed original articles, and provided quantitative data. Non-English publications, non-full text, and conference abstracts were excluded.

## 2. SARS-CoV-2 infection causes neurological symptoms

**Figure 1.**
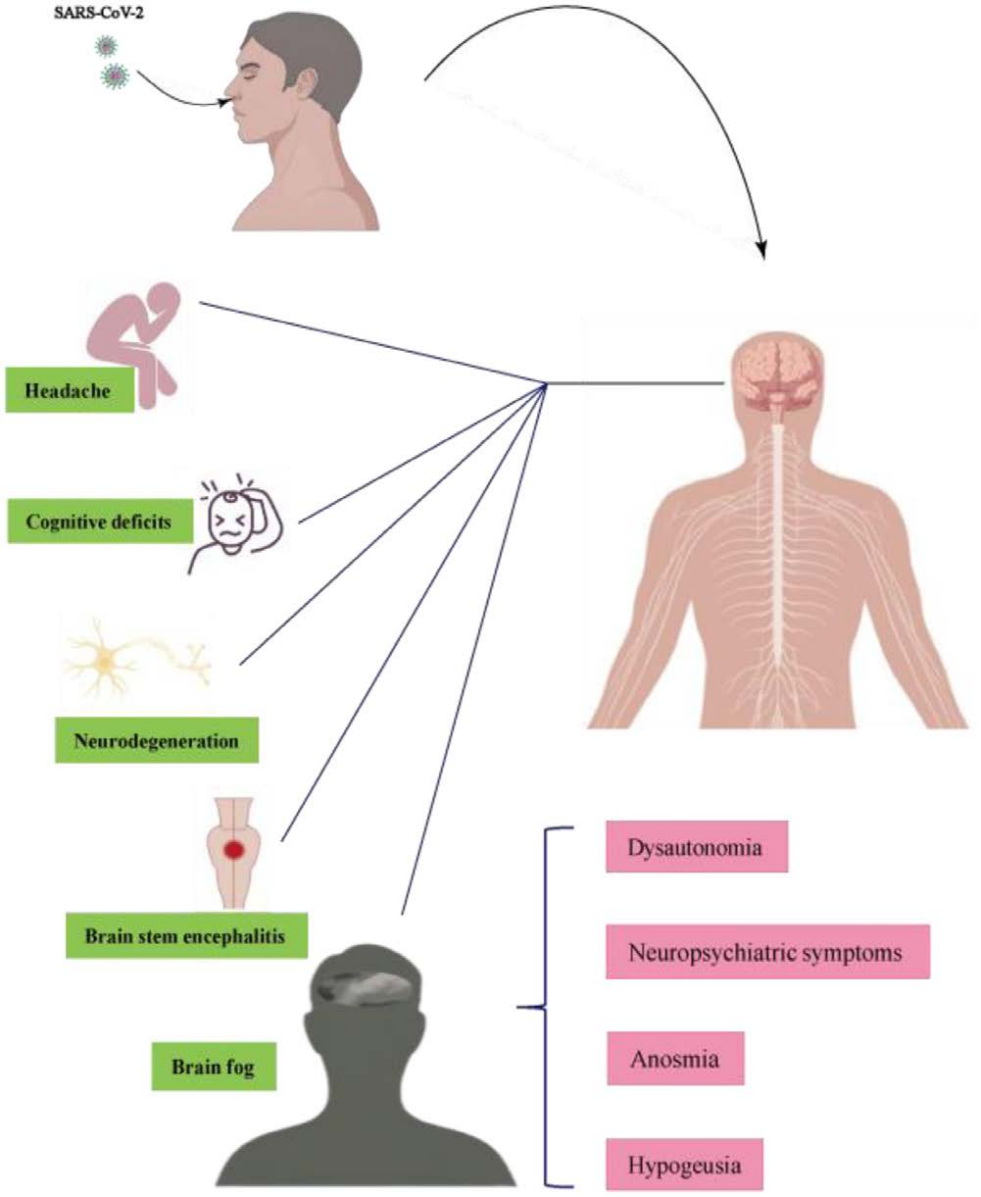
Neurological symptoms may occur in COVID-19 patients. After infection with SARS-CoV-2, the nervous system may be particularly affected, leading to neurological symptoms such as headache, cognitive deficits, neurodegeneration, brain stem encephalitis, and “brain fog”.

Several follow-up studies in patients suffering COVID have documented nervous system seems particularly affected after COVID-19 disease. In hospitalized patients with COVID-19, neurological symptoms occur in 8.8% to 57.4%.^[6]^ While many COVID-19 patients may exhibit mild neurologic symptoms, a small percentage of patients can develop severe neurologic disorders.^[1]^ The symptoms are mainly as follows: Headache is among the most frequently reported symptoms after resolution of COVID-19 and persistent headache after COVID-19 resolution could be an intermediate state between normality and migraine. 40 patients who recovered from COVID-19 but suffered from persistent headache without prior history of headache were assessed and found white matter changes were diffuse, involved most of the white matter tracts and seemed related to the impairment of white matter fiber bundles.^[7]^ Longitudinal studies revealed that cognitive deficits persist after 1-year follow-up ranging from 12%, 18% to 19% to 34% of COVID-19 patients. 86 patients with post-COVID syndrome were recruited and underwent neuroimaging acquisition and a comprehensive neuropsychological assessment and showed gray matter volume loss significantly associated with cognitive dysfunction.^[8]^ SARS-CoV-2 infection causes neurodegeneration. Age is the major risk factor for neurodegenerative diseases, and older patients present with more severe symptoms and prolonged course of COVID-19.^[9]^ Development and worsening of Alzheimers disease (AD), Parkinson disease, multiple sclerosis and most other common neurodegenerative diseases, have been associated with COVID-19.^[10]^ Sara et al performed a thorough literature screening and database mining to search for shared miRNA landscapes of SARS-CoV-2 infection and neurodegeneration and screened for miRNA targets identified 746 unique genes with strong evidence for interaction.^[11]^ Brain stem encephalitis is a rare complication of COVID-19. Out of thousands of hospitalized COVID-19 patients, only 8 patients were suspected to have central nervous system (CNS) symptoms directly related to COVID-19. However, in a small subset a clinically distinctive phenotype of brainstem encephalitis with cerebellar involvement is found. A direct link with SARS-CoV-2 is likely as increased antibody indices indicate specific intrathecal SARS-CoV-2 IgG production.^[12]^ In addition, the symptoms of long COVID can implicate persistent neurological complications in up to one third of patients and present as fatigue, “brain fog,” dysautonomia, neuropsychiatric symptoms, anosmia, hypogeusia, peripheral neuropathy and so on.^[13]^ The summary of neurological symptoms may occur in COVID-19 patients is shown in Figure 1. Altogether these studies indicate a relationship between SARS-CoV-2 infection and neurological conditions observed in COVID. And the neurological complications are associated with worse functional outcome, particularly in older subjects and those with comorbidities.^[14]^

## 3. SARS-CoV-2 causing the BBB dysfunction

As human coronaviruses, SARS-CoV-2 can target the CNS and cause damage by direct neurotoxicity or activation of the host immune response. CNS is an immune-privileged area. The blood-brain barrier (BBB) is thought to separate the CNS from any systemic inflammatory states to maintain homeostasis within this specialized, vulnerable organ. However, accumulating studies have challenged this concept by demonstrating systemic inflammatory effects on brain. Several lines of research suggested that breakdown to the integrity of the BBB and subsequent brain penetration of serum components and cytokines is responsible for the neurological manifestations after SARS-CoV-2 infection.^[15]^ The propensity of SARS-CoV-2 to cause the BBB dysfunction greatly increases the risk of chronic brain damage.^[16,17]^ The BBB is a highly selective dynamic interface composed of CNS ECs, astrocytes, pericytes, neurons, and microglia that form the neurovascular unit (NVU). BBB protects the brain from neurotoxins while allowing the supply of nutrients via solute carriers and the elimination of byproducts of brain metabolism via efflux transporters. BBB dysfunction underlies neurologic diseases responses to viruses triggered by pro-inflammatory.^[18,19]^ The main goal of this review is to summarize and elucidate damage to the main constituent cells of BBB, including BMECs, astrocytes and microglia and its contribution to COVID-19. Further understanding of these interactions may facilitate the development of improved treatment options and preventative measures of CNS injury due to COVID-19.

### 3.1. SARS-CoV-2 influencing brain microvascular endothelial cells

SARS-CoV-2 as an epitheliotropic virus, endothelial cell invasion is a common phenomenon. In COVID-19, the virus is thought to affect the BBB and lead to endothelial dysfunction which is a condition characterized by an impairment of the normal functioning of ECs, having important consequences, including ischemia, altered angiogenesis and coagulation, inflammation, and tissue edema.^[20,21]^ Viral particles in ECs may have implications for the route of entry of SARS-CoV-2 into the CNS, mainly including the hematogenous and neuronal retrograde pathways. In the hematogenous route, viruses gain access by infecting ECs of the BBB, epithelial cells of the blood-cerebrospinal fluid barrier in the choroid plexus, or use inflammatory cells as Trojan horses to gain access into the CNS. Alberto et al^[22]^ observed viral-like particles in the brain capillary endothelium and found actively budding across ECs strongly, suggesting a similar role of the endothelial bed and the hematogenous route as the most likely pathway for SARS-CoV-2 to reach the brain. In an in vitro BBB model, the SARS-CoV-2 envelope protein can bind to brain ECs and traverse the BBB, leading to damaging the BBB and inducing inflammatory responses in astrocytes. In addition, the inflammatory factors produced by brain ECs and astrocytes, in turn, may exacerbate BBB damage.^[23]^ SARS-CoV-2 may cross the BBB by invading BMECs and cause inflammation in brain. So how does SARS-CoV-2 enter BMECs? Brain tissues from 13 autopsies of people who died of COVID-19 were examined, meanwhile combining animal and cell experiments. It is found pseudovirions (spike, envelope, and membrane proteins without viral RNA) were present in the endothelia of microvessels and strongly co-localized with caspase-3, angiotensin-2 converting enzyme(ACE2), IL-6, tumor necrosis factor-α (TNF-α), and C5b-9, indicating ACE2 + endothelial damage is a central part of SARS-CoV-2 pathology.^[24]^ Brain ECs are susceptible to direct SARS-CoV-2 infection through flow-dependent expression of ACE2. Viral S-protein binding triggers a unique gene expression profile in brain endothelia.^[25]^ It is now known that SARS-CoV-2 infects human cerebrovascular endothelial cell, and ACE2 serves as an entry receptor to the virus, representing its main route of entry into the host cell.^[25]^ In addition to ACE2, SARS-CoV-2 infects human cerebrovascular endothelial cell also through Neuropilin-1, a transmembrane glycoprotein that has been implicated in several processes including angiogenesis and immunity and gangliosides are also an important entry point of SARS-CoV-2 into these cells.^[26,27]^ To understand the underlying mechanisms of viral transmigration through BMECs, human brain microvascular endothelial cells (HBMECs) were infected in vitro with SARS-CoV-2. Transcriptomic profiling of infected cultures revealed endothelial activation via NF-κB non-canonical pathway, including RELB overexpression, and mitochondrial dysfunction, confirming endothelial activation and remodeling can further contribute to neuroinflammatory processes and lead to further BBB permeability in COVID-19.^[21]^ Using the original strain of SARS-CoV-2, infected Human-induced pluripotent stem cells (iPSC) derived brain microvascular endothelial like cells (iPSC-BMELCs), caused permeability impairment and Wnt/β-catenin signaling is involved in SARS-CoV-2 infection of BMECs.^[28]^ Interestingly, SARS-CoV-2 infection triggered mast cells accumulation in the cerebrovascular region of mice and induced mast cells degranulation to disrupt the tight junction proteins in BMECs.^[29]^ In addition, diabetics are more vulnerable to SARS-CoV-2 neurological manifestations. In vitro, using HBMECs treated with high glucose-conditioned and using diabetic human angiotensin converting enzyme 2 (hACE2) mice in vivo infected by SARS-CoV-2 spike protein, found that SAR-CoV-2 spike protein intensified RAAS and TLR signaling in diabetes, increasing cerebrovascular damage and cognitive dysfunction.^[30]^

### 3.2. SARS-CoV-2 influencing astrocytes

Astrocytes are the most abundant glial cells in the CNS and display high functional heterogeneity. One of the important function of astrocytes is the maintenance of the BBB. Astrocytes interact with capillary ECs through their perivascular endfeet and help maintain the integrity of the BBB. In addition to microglia, astrocytes have emerged as key contributors to the innate immune response of the CNS to infections, neurodegenerative disorders, and injuries.^[31]^ Astrocytes form a parenchymal part of the BBB, the site of virus entry into the CNS. They exhibit aerobic glycolysis, a form of metabolism characteristic of highly morphologically plastic cells, like cancer cells, hence a suitable milieu for multiplication of infectious agent, including viral particles.^[32]^ It has been reported that SARS-CoV-2 can directly infect and damage astrocytes. A greater number of COVID-19 patients with a higher CFQ scores (a self-reported assessment of failures in perception, memory, and motor function, where the score in a healthy working population measures of a stable cognitive resource involved in attention, memory, and action in daily life at ten months) reflected cumulative neuronal and astroglial damage in the central nervous system.^[33]^ A potential ongoing injury affecting astrocytes following SARS-CoV-2 negativization, evident even ten months after the negativization.^[34]^ In primary cortical tissue cultures and cortical organoids exposed to SARS-CoV-2, it was observed significant infection and viral replication in immature and mature astrocytes, but minimal infection in other neural cell types and widespread inflammation, cytokine secretion, and reactivity in astrocytes as a response to infection.^[35]^ Based on SARS-CoV-2 viral RNA copies data, astrocytes were infected, and viral RNA copies are higher than pericytes following infection with the same amount of input virus.^[36]^ They are readily infected by SARS-CoV-2, however, cortical astrocytes do not express observable levels of ACE2, the canonical SARS-CoV-2 receptor, SARS-CoV-2 cofactors CD147 and DPP4 are highly expressed in infected astrocytes, whereas increasing expression of these receptors promoted infection, suggesting a role in viral entry or propagation.^[35]^ Other studies have found neuropilin-1, serves as the principal receptor mediating cell entry.^[35,37]^ Some changes occurred in astrocytes after infection. Astrocytes exhibited enlarged size and elevated nuclear fragmentation upon SARS-CoV-2 infection.^[38]^ Astrocyte infection produces a pathological response closely resembling reactive astrogliosis characterized by elevated type I interferon (IFN) production, increased inflammation, and the decreased expression of transporters of water, ions, choline, and neurotransmitters. These combined events initiated within astrocytes produce a hostile microenvironment that promotes the dysfunction and death of uninfected bystander neurons.^[37]^ Using astrocytes in vitro and dissected brain areas from SARS-CoV-2-infected Syrian hamsters to evaluate immunometabolic changes, it was shown an imbalance in important metabolic molecules and neurotransmitters in infected astrocytes, suggesting this may correlate with the neurological impairment observed during COVID-19, such as memory loss, confusion, and cognitive impairment.^[39]^ Astrocytes play important roles in mediating the tissue response to hypoxia-ischemia, which is the most common neuropathologic abnormality in COVID-19. Investigating the effects of SARS-CoV-2 on astrocytes in the inferior olivary nucleus, it was found neuropathology in COVID-19 is not due to hypoxia alone, and astrocytic pathology in COVID-19 may contribute to the prominent neuroinflammatory response in the brainstem.^[40]^

### 3.3. SARS-CoV-2 influencing microglia

CNS does not have a traditional lymphatic system or native lymphocytes. Microglia are brain-resident macrophages that regulate brain development, maintenance of neuronal networks and injury repair, also referred to as the brain’s immune cells. When microglia respond to immunological stimuli, they become activated and transform from a ramified into an ameboid morphology, releasing interleukin (IL)-1β, IL-6, and TNF-α.^[41,42]^ During homeostatic challenges to the brain parenchyma, microglia modify their density, morphology, and molecular signature, resulting in the adjustment of their functions.^[43]^ SARS-CoV-2 can directly infect human microglia and microglia are potential mediators of SARS-CoV-2-induced neurological problems. It was reported that autopsy of a 73-year-old man who died from acute cerebellar hemorrhage in the context of relatively mild SARS-CoV-2 infection, performed 3 hours after death showed cerebellar hemorrhage and acute infarcts in the dorsal pons and medulla. Remarkably, there were microglial nodules and neuronophagia bilaterally in the inferior olives and multifocally in the cerebellar dentate nuclei.^[44]^ The colocalization of the SARS-CoV-2 nucleocapsid within microglia by using confocal immunofluorescence in 3 deceased COVID-19 cases, of between 78 and 85 years of age at death.^[45]^ Microglia infected by SARS-CoV-2 perform pro-inflammatory functions in the CNS. Jeong et al^[46]^ reported that SARS-CoV-2 infect human microglia, eliciting M1-like proinflammatory responses, followed by cytopathic effects. Specifically, SARS-CoV-2 infected human microglial clone 3 (HMC3), leading to inflammatory activation and cell death. BV-2 microglia were activated by SARS-CoV-2 spike S1 protein, resulting in the production of pro-infammatory mediators TNFα, IL-6, IL-1β, and NO through activation of NF-κB and p38 MAPK, possibly as a result of TLR4 activation.^[41]^ Moreover, the microglial hyperactivation in the brainstem, and in the hippocampus of COVID-19 patients with delirium, appears as a specific topographical phenomenon, and probably represents the neuropathological basis of the “COVID-19 encephalopathic syndrome” in the elderly.^[47]^ Some studies have also been done on the mechanism of microglia activation in COVID-19. Matrix metalloproteinase-9 (MMP-9) can disrupt neuronal connectivity and be elevated in patients with long COVID. MMP-9 from Spike protein-stimulated microglia could contribute to the development of long COVID.^[48]^ SARS-CoV-2 Spike protein modulates microglial purinergic signaling in cultured microglial cells exposed to Spike protein, consistent with in hippocampal tissue of Spike infused animals, which may be a central event in COVID-19 neuropathology.^[49]^ However, there are different voices. It was reported that microglia activity decreased and apoptosis occurred after infection with SARS-COV-2. Using histological immunohistochemical studies of brain autopsy materials of 18 patients who had died from COVID-19, indicate a reduced activity of microglia.^[50]^ SARS-COV-2 spike protein increases the levels of pro-inflammatory cytokines and ROS production, increases apoptosis and increases the oxygen consumption rate in microglial cells.^[51]^ Additionally, Seehusen et al^[52]^ with SARS-CoV-2 variants infected transgenic mice expressing hACE2, microglial activation, microgliosis was consistently observed, accompanied by apoptotic death of microglial, without their apparent infection.

In conclusion, SARS-CoV-2 can directly infect BMECs and astrocytes through the corresponding receptors on the cells. And microglia, due to the CNS being infected by the virus, triggers an immune response and activates them. Here we present a hypothesis that SARS-CoV-2 may first enter the brain through the portal BMECs, then infect astrocytes, trigger an immune response in the CNS, and subsequently activate microglia to produce a neuroinflammatory response. However the responses of these cells to viral infection are not independent, instead, they interact with each other through cell-to-cell communication and contributing to inflammatory cascade causing neuro-inflammatory in the CNS.

## 4. Cross-talk between cells of the NVU

The BBB, which is mainly formed by ECs, represents a barrier interface between the systemic circulation and the CNS. Surrounding the microvessels and coordinating function with ECs are pericytes, astrocytes, neurons, and microglia forming functional elements of the BBB called the NVU. The barrier properties of the BBB are generated by high-resistance interendothelial tight junctions formed by transmembrane proteins, such as claudin-5, and cytoplasmic proteins, such as zonula occludens (ZO-1), that limit the paracellular permeability between ECs.^[53]^ Neurological complications are common in patients affected by COVID-19 due to the ability of SARS-CoV-2 to infect brains. While the mechanisms of this process are not fully understood, it has been proposed that SARS-CoV-2 can infect the cells of the NVU, which form the BBB. The receptors involved in SARS-CoV-2 infection are co-expressed in astrocytes and microglial cells. These receptors are functionally active as exposure of ECs to the SARS-CoV-2 S1 protein subunit altered the expression pattern of tight junction proteins, such as claudin-5 and ZO-1, which elucidate a possible pathway of the virus entry into the brain.^[53]^ SARS-CoV-2 concern or interest on the functional activities of astrocytes, BMECs and microglia, it suggests that SARS-CoV-2 is neurotropic, with deleterious consequences for the BBB integrity and central nervous system cells, which could underlie neurological disorders following SARS-CoV-2 infection.^[54]^ Importantly, the cells that make up the NVU have cross-talk with each other, influencing each other during viral infection. The secretome of primary HBMECs, obtained after exposure to the S1 subunit of the SARS-CoV-2 spike (S) protein activated STAT3 in microglial cells, indicating that proinflammatory signals from ECs can propagate to other cells of the NVU.^[55]^ In brief, SARS-CoV-2 infects CNS through BBB and causes breakdown of BBB to promote neuroinflammatory response, which is the basis of various neuropsychiatric symptoms. However, at present, the interactions of ECs, astrocytes and microglia when SARS-CoV-2 infects with the NVU are largely unknown. Exosomes (EXs), a type of extracellular vesicle (EV) of cell-derived nanoparticles harboring various cellular components such as proteins, mRNAs, and microRNAs (miRs), have been shown to be an effective means of cell-to-cell and organ-to-organ communication by delivering their cargoes. Roles of EXs as intercellular communicators have been extensively studied in the last few decades, especially under various pathological conditions.^[56]^ As each cell of the NVU can produce EXs that could help in the communication between cells in short or long distances. EXs are able to cross the BBB from the brain to the bloodstream as well as from the blood to the CNS.^[57]^ In the context of viral infections, EXs can also contain viruses, viral proteins, and viral nucleic acids. Packaging of viruses within EXs has repeatedly been shown to protect viruses from antibody neutralization while also allowing for their integration into cells otherwise impervious to the virus.^[58]^ To sum up, in the CNS, cross-talk between cells of the NVU is crucial for a variety of biological functions, ranging from brain development, neural circuit maturation, homeostasis maintenance. Next, we focus on current knowledge and potential relation regarding EXs associated with cross-talk between the cells of the NVU during virus infection and other pathological processes.

In HIV infection, HIV-infected brains are characterized by increased amyloid beta (Aβ) deposition. HIV-1 hijacks the exosomal pathway for viral budding and release. HIV-1 increases the release of EVs/EXs from BMECs and elevates their Aβ cargo. Interestingly, brain endothelial cell-derived EVs/EXs -Aβ cargo can be successfully transferred to astrocytes and pericytes. It was suggested that EVs/EXs carrying Aβ can be successfully transferred across the BBB into the brain and microvascular endothelium derived EVs/EXs enables a cross-talk between the 2 cell types and are known to induce the BBB phenotype.^[59]^ Microglia become persistently infected during Theiler murine encephalomyelitis virus infection in the CNS of susceptible mice. The EXs secreted by microglia during Theiler murine encephalomyelitis virus infection which contain the viral RNA coding region can be taken up by uninfected bystander cells, including microglia, astrocytes, and neurons and the viral RNA transferred to the bystander cells. Then, the bystander cells that took up these EXs were activated to promote an inflammatory immune response.^[60]^ In terms of bacterial infections, Yang et al^[61]^ through in vivo and in vitro models demonstrated that a total of 57 proteins were significantly differentially expressed in the EXs secreted by BMECs infected with Escherichia coli. Among them, growth differentiation factor 15 significantly increased in the EXs secreted by BMECs during infection, which triggered the Erk1/2 signaling pathway and promoted the activation of astrocytes. In cerebral ischemia and brain injury diseases, EXs also play a significant role in mediating the information transmission between cells of the NVU. Oxygen-glucose deprivation-cultured BMECs secrete miR-3613-3p to transfer into microglia via EXs, and miR-3613-3p binds to the ring finger and CCCH-type domains 1 (*RC3H1*) 3’ untranslated region to reduce RC3H1 protein levels in microglia. Exosomal miR-3613-3p promotes microglial M1 polarization by inhibiting RC3H1 protein levels. BMEC exosomal miR-3613-3p reduces neuronal survival by regulating microglial M1 polarization.^[62]^

Recent studies have shown that EXs derived from activated astrocytes promote microglial M2 phenotype transformation following miRNAs in the EXs derived from activated astrocytes play a key role in the astrocyte-microglia interaction during traumatic brain injury.^[63]^ In a rat traumatic brain injury model, astrocyte-derived exosomal miR-148a-3p regulates the microglial phenotype, shifting it from the M1 phenotype to the M2 phenotype, inhibits neuroinflammation, and restores neurological function.^[64]^ The etiologies and origins of neurodegenerative diseases, such as AD, Parkinsons disease (PD), and Huntingtons disease, are complex and multifaceted. A growing evidence suggests that EXs play an important role in the occurrence and development of neurodegenerative diseases.^[65]^ Dopaminergic neurons internalize astrocyte-secreted EXs and that LRRK2 G2019S (PD-related mutation) EXs are abnormally enriched in neurites and fail to provide full neurotrophic support to dopaminergic neurons. Thus, dysfunctional astrocyte-to-neuron communication via altered EXs biological properties may participate in the progression of PD.^[66]^ In addition, exploring the differences in gene expression, it was found that astrocyte EXs affect neurodegenerative diseases mainly through metabolic balance and ubiquitin-dependent protein balance. EXs derived from cholesterol-accumulated astrocytes can play an important role in trafficking APP/Aβ peptides and influencing neuronal viability in the affected regions of the AD brain.^[67]^ These findings showed that EXs act as potent mediators involved in intercellular communication in the NVU regardless of virus infection, brain injury or neurodegenerative diseases and so on, suggesting that the cells of NVU interact with each other during the development of the disease.

## 5. Conclusion

**Figure 2.**
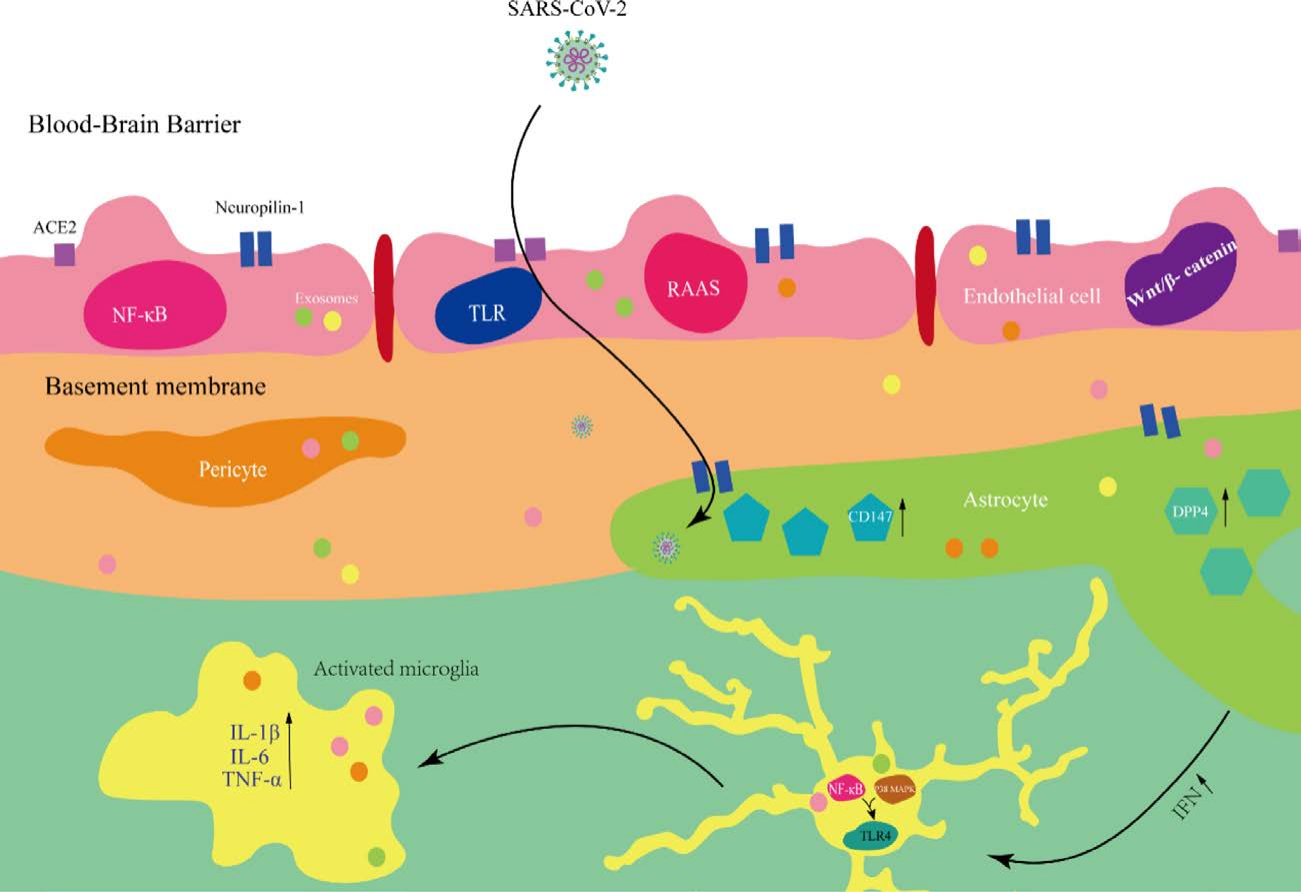
SARS-CoV-2 causes blood-brain barrier dysfunction. The blood-brain barrier is primarily composed of endothelial cells and is surrounded by astrocytes, pericytes, neurons, and microglia. These components coordinate with endothelial cells to form the functional units of the blood-brain barrier. They can produce EXs, facilitating short or long distances communication between cells. SARS-CoV-2 can directly infect BMECs via receptors such as ACE2 and Neuropilin-1, leading to blood-brain barrier disruption. Additionally, it can induce inflammatory responses in astrocytes, triggering microglial activation in response to immune stimulation, resulting in morphological changes and the release of inflammatory factors such as IL-1β, IL-6, and TNF-α.

The neurological symptoms are not specific to COVID-19 patients. Similar symptoms have been observed in some cases of infection via other viruses, such as herpes simplex, HIV, Chikungunya, Dengue, and Zika virus, although the tropism of coronaviruses is not specific to the CNS.^[68]^ The pathophysiology behind neurological and cognitive sequelae of COVID-19 related to transient increased BBB permeability and neuronal injury and glial activation.^[69–71]^ Its mechanism is closely related to EXs-mediated intercellular interaction across the BBB (Fig. 2). Therefore, the treatment strategies of the neurological symptoms caused by SARS-CoV-2 could be considered focus on blocking viral invasion, regulating EXs functions, and inhibiting neuroinflammation: Blocking the direct attack of the virus on the BBB: Inhibiting the entry of the virus into BMECs, such as using ACE2 receptor antagonists and antibodies against viral S-protein and enhancing Tight Junction Proteins to maintaining BBB Integrity. Intervening EXs-mediated pathological signals: Elimination or neutralization of pathogenic EXs using EXs capture technology, and engineering exosomes to carry siRNA or miRNA (such as siRNA targeting IL-6, NLRP3, or anti-inflammatory miR-146a) to precisely inhibit pro-inflammatory signaling pathways. Inhibiting neuroinflammation and excessive activation of glial cells: By loading anti-inflammatory molecules (such as IL-6 inhibitors, TNF-α antagonists) onto engineered EXs, and using the natural chemotaxis of EXs to target and deliver them to the brain, the excessive activation of glial cells (microglia, astrocytes) can be inhibited.

SARS-CoV-2 viral infection caused a global panic and a scientific push to develop a cure. According to the published literature, neurological complications appear to be more debilitating than complications reported in other organ systems in COVID-19. Clarifying various mechanisms leading to the development of the neurological manifestations would improve the ability of physicians to treat patients and of researchers to develop a drug to target the disease state.^[72,73]^ Therefore, identifying the mechanisms of BBB damage caused by COVID-19 in order to avoid the virus perverting the host response is an important task, and that should be carried out in order to protect humankind against constantly mutating SARS-CoV-2 in the future.

## Author contributions

**Conceptualization:** Jin Li.

**Data curation:** Shangfu Xu, Sixu Guo.

**Funding acquisition:** Jin Li, Shangfu Xu.

**Visualization:** Jin Li.

**Writing – original draft:** Jin Li.

**Writing – review & editing:** Shangfu Xu, Sixu Guo.
